# Comparison of Perineal Sonographically Measured and Functional Urodynamic Urethral Length in Female Urinary Incontinence

**DOI:** 10.1155/2016/4953091

**Published:** 2016-10-13

**Authors:** Laila Najjari, Nadine Janetzki, Lieven Kennes, Elmar Stickeler, Julia Serno, Julia Behrendt

**Affiliations:** ^1^Department of Gynecology and Obstetrics, University Hospital RWTH Aachen, Aachen, Germany; ^2^Institute of Medical Statistics, University Hospital RWTH Aachen, Aachen, Germany

## Abstract

*Objectives*. To detect the anatomical insufficiency of the urethra and to propose perineal ultrasound as a useful, noninvasive tool for the evaluation of incontinence, we compared the anatomical length of the urethra with the urodynamic functional urethral length. We also compared the urethral length between continent and incontinent females.* Methods*. 149 female patients were enrolled and divided into four groups (stress, urge, or mixed incontinence; control). Sonographically measured urethral length (SUL) and urodynamic functional urethral length (FUL) were analyzed statistically. Standardized and internationally validated incontinence questionnaire ICIQ-SF results were compared between each patient group.* Results*. Perineal SUL was significantly longer in incontinent compared to continent patients (*p* < 0.0001). Pairwise comparison of each incontinent type (stress, urge, or mixed incontinence) with the control group showed also a significant difference (*p* < 0.05). FUL was significantly shorter in incontinent patients than in the control group (*p* = 0.0112). But pairwise comparison showed only a significant difference for the stress incontinence group compared with the control group (*p* = 0.0084) and not for the urge or mixed incontinent group. No clear correlation between SUL, FUL, and ICIQ-SF score was found.* Conclusions*. SUL measured by noninvasive perineal ultrasound is a suitable parameter in the assessment of female incontinence, since incontinent women show a significantly elongated urethra as a sign of tissue insufficiency, independent of the type of incontinence.

## 1. Introduction

Urinary incontinence in females has an increasing prevalence [1, 2]. Its diagnosis is mostly possible with a thorough history taking and clinical evaluation. But additional invasive urodynamic investigations are often necessary and help to further classify the type of incontinence and to facilitate the preoperative planning [3, 4]. One relevant parameter of the urodynamic investigation is the functional urethral length (FUL). There are a huge number of publications from the eighties on the FUL all showing that the mean length was lower in incontinent (especially stress incontinent) versus continent women [5, 6]. But still, up until now, its usefulness for establishing a precise diagnosis of urinary incontinence is debated, since measurements are often affected by artefacts and the overlap of values between continent and incontinent women was huge.

Besides urodynamic measurements, other modalities like magnetic resonance imaging (MRI) or dynamic cystocolpoproctography (DCP) are used for further examination of incontinence, especially giving further objective anatomical information like bladder position. But these techniques are either partly invasive, laborious and embarrassing for the patient, or expensive diagnostic tools with long waiting periods.

In contrast, perineal ultrasound (PUS) is gaining importance in urogynecological diagnostics. Good availability, easy handling, low cost, and good patient acceptance are some of the conveniences which have already made PUS a popular diagnostic tool, for example, in the assessment of pelvic organ prolapse, the detection of paraurethral pathologies, and the postoperative sonographic control of tension-free vaginal tape (TVT) slings [7]. But even with these benefits, the use of PUS is not as widespread as it could be and especially the role of PUS in the diagnostic of incontinence is still unclear, although it may contribute great advantages. Therefore, the present study was intended to investigate the potentials of PUS as a noninvasive diagnostic tool in incontinence. For this, it was analyzed whether the anatomical urethral length measured by PUS is a good diagnostic parameter to assess female urinary incontinence.

## 2. Materials and Methods

The present study was performed according to the Declaration of Helsinki and with approval of the local ethics committee (reference number EK085/11).

As a new approach to diagnosing incontinence, we assigned this study to the development stage of the IDEAL method (Stage 2a) [8]. Therefore, to investigate the potential of PUS, we chose to perform PUS on a small collective of patients with only one examiner in our center.

### 2.1. Patients

All 149 women who presented at our continence center between 2008 and 2012 were retrospectively included. Data acquisition was performed using an electronically data program which continuously documented all patients. Patient data included patients' history and results of clinical examinations, complete urodynamic investigations, and perineal ultrasonography. Study patients (Table 1) were divided into three groups: Patients in group I suffered from stress urinary incontinence (SUI), patients in group II from urgency urinary incontinence (UUI), and patients in group III from both stress and urgency urinary incontinence (SUI/UUI). A control group (group IV) consisted of patients who clinically and urodynamically showed no criteria of urinary incontinence.

### 2.2. Urodynamic Investigation: FUL

All urodynamic investigations were performed according to the description by Schaefer et al. 2002 [4]. For urodynamic investigation of the FUL, the urethra pressure profile was evaluated with a 40 cm long three-lumen catheter CAT307 (Laborie, Mississauga, Canada) and the pressure sensor (Transducer) MX960XP1 (Smith Medical International LTC, St. Paul, USA). While retracting (0.7 cm/s) the catheter mechanically under a constant saline perfusion rate (2–10 mL/min), intravesical and intraurethral pressure were measured simultaneously. FUL then is defined as the distance in which the intraurethral pressure exceeds the intravesical pressure.

### 2.3. Perineal Ultrasonography: SUL

**Figure 1:**
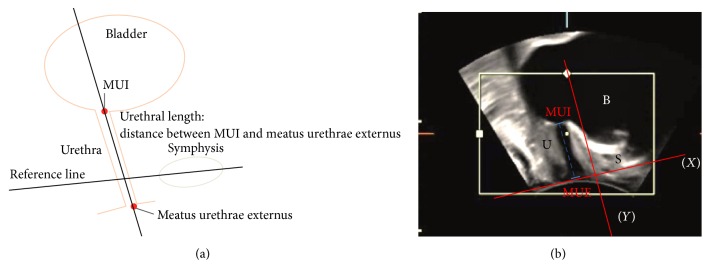
(a) Schematic illustration of bladder and measurement of urethral length (MUI: meatus urethrae internus). (b) Ultrasound image showing bladder (B), symphysis (S), meatus urethrae internus (MUI), and transperineal measurement of the sonographic urethral length (SUL).

PUS was performed by an experienced board qualified gynecologist according to the DEGUM Level II standard (Deutsche Gesellschaft für Ultraschall in der Medizin) [9]. Patients were asked to drink two glasses of water half an hour prior to the examination to reach a bladder filling of approximately 300 mL. Ultrasonography was performed with the patient in the lithotomy position using a Voluson 730 Expert (GE Health Care, Wauwatosa, USA) with a 3.5 to 5 MHz transperineal probe (GE Medical Systems, Zipf, Austria) [10]. After covering the transducer with a condom, the examiner parted the labia and placed the transducer on the perineum [10]. Sagittal pictures were obtained according to a standard protocol as shown in Figures 1(a) and 1(b). Patients were asked to rest and then to perform the following maneuvers: pelvic floor muscle contraction, Valsalva maneuver, and coughing. A four-dimensional video volume of the ultrasound evaluation was recorded for each patient. Analysis of the data was performed later, using the software 4DView (GE Medical Systems, Systems, Zipf, Austria) and the sonographic urethral length at rest (SUL-R), during contraction (SUL-C) and under pressure (SUL-P), was determined.  Figure 1(b) demonstrates a measurement of the urethra length as shown by the punctuated linear line from the intraurethral opening to the external opening of the urethra.

### 2.4. Clinical Questionnaire: ICIQ Score

The standardized and internationally validated incontinence questionnaire ICIQ-SF (International Consultation on Incontinence Questionnaire-Short Form) as a measure for the psychological strain of incontinent was obtained from the patients during the first visit. The score ranges from zero to 21, with zero being no strain at all.

### 2.5. Statistical Analysis

An explorative data analysis was performed with the significance level at *p* ≤ 0.05. Statistical analysis was performed with Med Calc version 9.2.0.1 (Ostend, Belgium).

SUL and FUL values for all patient groups were given as median, mean, maximum, and minimum as well as quartiles and interquartile distance and standard deviation. Comparison of the FUL was performed by Mann–Whitney* U* test. Analysis of the SUL values was performed by* t*-test and Welch-Test. For the correlation of SUL-R, FUL, and ICIQ scores, Spearman's rank correlation coefficient* r* was calculated. The higher the correlation between parameters, the closer the correlation coefficient* r* to −1 (antiproportional correlation) or +1 (proportional correlation).

## 3. Results

### 3.1. Patients

149 women were included in the study. 117 of the patients were diagnosed with incontinence: 72/117 (61.5%) with stress urinary incontinence (SUI), 22/117 (18.8%) with urgency urinary incontinence (UUI), and 23/117 (19.65%) with both, called mixed urinary incontinence (SUI/UUI). Median age of the incontinent women was 62 ± 11 (35–83), 67 ± 10 (49–82), and 65 ± 12 (41–90) years for the stress, urge, and mixed incontinent patients groups, respectively. The female control group consisted of 32 patients with a mean age of 62 ± 12 years (range 41–80 years).

### 3.2. FUL

FUL values for the three incontinent patient groups (SUI, UUI, and SUI/UUI) and the control group are given in Table 1(a). Mean and median value of the incontinent women were shorter compared to the control group. Statistical significance was reached between incontinent patients and the control group (*p* = 0.0112). Subsequent pairwise* t*-tests between each incontinent group with the control group showed only a significant difference for the SUI group compared with the control group (*p* = 0.0084). No significant difference was found comparing the incontinent patients groups UUI and SUI/UUI with the continent group (Table 1(b)). Furthermore, pairwise comparison of the three types of incontinence (SUI, UUI, and SUI/UUI) showed no significant difference (Table 1(b)).

### 3.3. SUL

SUL values for the three incontinent patient groups (SUI, UUI, and SUI/UUI) and the control group under the three conditions (rest, contraction, and pressure) are given in Tables 2(a)–4(a).

SUL-R was statistically highly significantly longer in all incontinent patients compared to the continent control patients (*p* < 0.0001). Subsequent pairwise comparison of each incontinent type (SUI, UUI, and SUI/UUI) with the control group showed also a statistically significant difference (Table 2(b)).

SUL-P values were statistically highly significantly longer in all incontinent patients compared to the continent control patients (*p* < 0.0001). Subsequent pairwise comparison of the incontinent patients with the control group showed also a statistically significant difference independent of the type of urinary incontinence (Table 3(b)).

Similar results were seen comparing the SUL-C results. Values were statistically significantly longer in all incontinent patients compared to the continent control patients (*p* < 0.003). Pairwise comparison of the incontinent patients with the control group showed also a statistically significant longer SUL-C for the SUI and the SUI/UUI groups. Comparison of the UUI with the control group revealed no statistically significant difference (*p* = 0.2618) (Table 4(b)).

Spearman's rank correlation in incontinent patients between the FUL and the SUL-R, SUL-P, and SUL-C showed only a very weak correlation with a coefficient of *r* = −0.064, *r* = 0.05, and *r* = 0.077, respectively.

### 3.4. ICIQ Score

The ICIQ score was obtained in 110 of the incontinent patients with a mean score of 13.8 ± 4.5. The mean ICIQ score of the SUI (*n* = 69), UUI (*n* = 21), and SUI/UUI (*n* = 20) group was 14.4 ± 3.7 (range 4–21), 11.8 ± 6.2 (range 0–21), and 14 ± 4.5 (range 6–21), respectively.

Analysis of Spearman's rank correlation between the ICIQ score and the FUL, the SUL-R, SUL-P, and SUL-C showed a coefficient of *r* = −0.124, *r* = 0.026, *r* = 0.356, and *r* = 0.182, respectively.

In summary, no clear correlation between ICIQ data and FUL or SUL was found.

## 4. Discussion

The prevalence of female urinary incontinence is up to 25% depending on age [11]. The diagnosis of urinary incontinence follows an accurate case-history collection including standardized questionnaires. There is an ongoing effort to correlate clinical symptoms with objective measurements, for example, the urethral pressure profile established by urodynamic investigations [12]. Measurement parameters include maximum urethral closure pressure, active and passive pressure transmission, and the functional urethra length [4, 12]. Still, their clinical relevance is debatable and the investigations are partly invasive, laborious, and embarrassing for the patient. In contrast, perineal ultrasound has gained importance in urogynecological diagnostics for it is easy to handle, good, available, and of low cost. Furthermore, it easily provides additional diagnostic information, for example, about pelvic organ prolapse or paraurethral pathologies [10, 13]. This clinical study aimed to evaluate whether the anatomical urethral length measured by perineal ultrasound can serve as a useful diagnostic tool in assessing urinary incontinence in women.

Our study clearly demonstrates that the perineal sonographically measured urethral length differs statistically significantly between continent and incontinent females with a statistically significant longer SUL value in incontinent patients. The difference is best seen in the examinations at rest and under pressure and least during pelvic muscle contraction. Under these two conditions, the SUL was statistically significantly longer for the stress, urge, and mixed incontinence group. Urinary incontinence has multifactorial causes such as age, child birth, and insufficiency of the connective tissue. As urinary incontinence is often associated with a genital prolapse [14], we assume that insufficiency of the urethral tissue itself may be the reason for the longer anatomical urethral length. Up until now, there have been quite a few reports about the association of urethral hypermobility with urinary incontinence [15, 16]. However, there is hardly any literature about a possible association between urethral elongation and urinary incontinence. It has to be assumed that the reproducibility is best in an examination at rest because investigations under pressure or during contraction are influenced and potentially falsified by patient related factors and thus hardly to reproduce precisely. Furthermore, the FUL is only evaluated at rest and thus a direct comparison of FUL and SUL under the same conditions is ensured. Therefore, we recommend measuring the sonographic urethral length at rest. SUL could be shorter in continent women due to compression by the examiner, better contractility of intact pelvic muscles, or unconscious tension.

Results show that there is no statistical significant difference of SUL between the three types of incontinence; therefore, PUS cannot help to clearly differentiate between the three different types of incontinence. However, the longest average SUL at rest is observed in patients with stress urinary incontinence (3.85 cm ± 0.68 cm) compared to the shortest urethral length in patients with urgency urinary incontinence (3.63 cm ± 0.72 cm). The average urethral length in continent patients is 2.87 cm ± 0.38 cm. Reasons for the elongated urethra especially in females with stress urinary incontinence are anatomical changes with generalized pelvic floor insufficiency, vaginal deliveries, and age [17, 18]. Future larger studies have to show at what cut-off value SUL can serve as a reliable diagnostic tool in the assessment of incontinence.

In contrast to the noninvasive SUL measurements, results from the urodynamic FUL showed only a statistically significant difference for the stress incontinent compared to the control group. These findings are in accordance with previous studies reporting from a reduced FUL in stress incontinent patients [5, 6, 19]. Analysis for the urge and mixed incontinent patients revealed no statistically significant shorter FUL and consequently these two types of incontinence cannot be detected by FUL measurement. In addition, FUL cannot differentiate between the different types of urinary incontinence, for there was no statistically significant difference between the different incontinent patient groups.

The comparison of the SUL at rest with the urodynamically measured FUL showed no correlation and even no antiproportional correlation as one might expect. But FUL and SUL are completely different parameters, as FUL describes a urodynamic functional finding and SUL an anatomical finding. Thus, taking this into account, these findings seem to be comprehensible.

The degree of urinary incontinence is difficult to determine but can be estimated, for example, with the subjective questionnaire tool of the ICIQ. According to Karantanis et al., the score correlates with the degree of urinary incontinence and is recommended as measurement tool [20]. In our study, correlation between the ICIQ values and SUL indicates a correlation of high ICIQ values and longer anatomical urethral length, but values show quite a lot scattering around the regression line and statistically there was no correlation found. The same phenomenon was seen when correlating the objectively measured FUL with the ICIQ results: results only show a tendency of increased ICIQ values with shortened functional urethra length. In summary, no statistically significant correlation is seen neither with functional nor with anatomical length and ICIQ scores.

Our study has quite a few limitations. As we only have a small number of patients, we cannot make definite conclusions concerning the statistical differences between both groups. However, we have statistically significant results. This can encourage further studies with the possibility of establishing a larger control group of healthy population, which is needed to obtain results referring to a normal distribution. As this is a stage 2a study, we were limited to the patients in our hospital who came to us with the diagnosis of urinary incontinence. Because of the limitation of only being able to perform urodynamics with our patients, we have an inhomogeneous age distribution. Also, only one examiner performed one examination; therefore, the repeatability cannot be assessed. Further, probable causes for a lack of correlation might be the limited number of patients, and therefore it is possible that larger scale studies may find a correlation between ICIQ and SUL or FUL. Another limitation might be that the control group was recruited from our gynecological clinic and some women, though incontinence was excluded by clinical and urodynamic evaluation, showed minor signs of pelvic insufficiency. This may also have influenced both the questionnaire and the objective measurements.

## 5. Conclusions

We believe to have obtained interesting results which should be pursued further in order to gain a better insight into the pathophysiology of urinary incontinence, as well as gaining a new parameter in the assessment of female incontinence. In this study, SUL measured by perineal ultrasound was a suitable parameter to differentiate between continent and incontinent females independently of the three types: stress, urge, and mixed incontinence. In incontinent females, a statistically significant elongated urethra was found. In contrast, the parameter FUL was only statistically significant altered in stress but not in urge or mixed incontinent patients compared to the control group. Furthermore, perineal ultrasound provides the advantage of a noninvasive tool compared to the invasive urodynamic investigations and additionally facilitates the evaluation of comorbidities such as urethral kinking or funneling, obstruction, or paraurethral pathologies [21–23]. Thus, further studies with focus on perineal SUL measurement should be considered in patients with presumed incontinence.

## Figures and Tables

**(a):** 

Group	Mean (mm)	SD	Median (mm)
SUI	22.44	7.48	20
UUI	21.95	6.32	21.5
SUI/UUI	22.61	6.13	22
Control	24.22	6.47	25

**(b):** 

Groups	*p*
SUI versus control	0.0084
SUI versus UUI	0.6265
SUI versus SUI/UUI	0.4980
UUI versus control	0.1369
UUI versus SUI/UUI	0.9186
SUI/UUI versus control	0.1246

**Table:** (a) FUL

Group	Mean (cm)	SD	Median (cm)
SUI	3.85	0.68	3.91
UUI	3.63	0.72	3.68
SUI/UUI	3.82	0.65	3.76
Control	2.87	0.38	2.84

**(b):** 

Groups	*p*
SUI versus control	0.0001
SUI versus UUI	0.1960
SUI versus SUI/UUI	0.8562
UUI versus control	0.0001
UUI versus SUI/UUI	0.3560
SUI/UUI versus control	0.0001

**Table:** (a) FUL

Group	Mean (cm)	SD	Median (cm)
SUI	3.18	0.85	3.18
UUI	3.05	0.87	2.92
SUI/UUI	3.37	0.77	3.45
Control	2.13	0.57	2.17

**(b):** 

Groups	*p*
SUI versus control	0.017
SUI versus UUI	0.5278
SUI versus SUI/UUI	0.3536
UUI versus control	0.0001
UUI versus SUI/UUI	0.2005
SUI/UUI versus control	<0.0001

**Table:** (a) FUL

Group	Mean (cm)	SD	Median (cm)
SUI	3.45	0.55	3.34
UUI	3.35	0.55	3.31
SUI/UUI	3.62	0.54	3.56
Control	3.20	0.40	3.23

**(b):** 

Groups	*p*
SUI versus control	0.0093
SUI versus UUI	0.4221
SUI versus SUI/UUI	0.2094
UUI versus control	0.2618
UUI versus SUI/UUI	0.0988
SUI/UUI versus control	0.0016
